# The induction of activating transcription factor 3 (ATF3) contributes to anti-cancer activity of *Abeliophyllum distichum* Nakai in human colorectal cancer cells

**DOI:** 10.1186/1472-6882-14-487

**Published:** 2014-12-15

**Authors:** Gwang Hun Park, Jae Ho Park, Hyun Ji Eo, Hun Min Song, So Hee Woo, Mi Kyoung Kim, Jin Wook Lee, Man Hyo Lee, Jeong Rak Lee, Jin Suk Koo, Jin Boo Jeong

**Affiliations:** Department of Bioresource Sciences, Andong National University, Andong, 760749 Korea; Department of Medicinal Plant Science, Jungwon University, Goesan, 367805 Korea; Gyeongbuk Institute for Bio-industry, Andong, 760380 Korea; Insititute of Agricultural Science and Technology, Andong National University, Andong, 760749 Korea; Department of Medicinal Plant Resources, Andong National University, Andong, 760749 Korea

**Keywords:** *Abeliophyllum distichum* Nakai, Activating transcription factor 3, Apoptosis, Cancer chemoprevention, Colorectal cancer

## Abstract

**Background:**

Recently, *Abeliophyllum distichum* Nakai (*A. distichum*) has been reported to exert the inhibitory effect on angiotensin converting enzyme. However, no specific pharmacological effects from *A. distichum* have been described. We performed *in vitro* study to evaluate anti-cancer properties of *A. distichum* and then elucidate the potential mechanisms.

**Methods:**

Cell viability was measured by MTT assay. ATF3 expression level was evaluated by Western blot or RT-PCR and ATF3 transcriptional activity was determined using a dual-luciferase assay kit after the transfection of ATF3 promoter constructs. In addition, ATF3-dependent apoptosis was evaluated by Western blot after ATF3 knockdown using ATF3 siRNA.

**Results:**

Exposure of ethyl acetate fraction from the parts of *A. distichum including* flower, leaf and branch to human colorectal cancer cells, breast cancer cells and hepatocellular carcinoma reduced the cell viability. The branch extracts from *A. distichum* (EAFAD-B) increased the expression of activating transcription factor 3 (ATF3) and promoter activity, indicating transcriptional activation of ATF3 gene by EAFAD-B. In addition, our data showed that EAFAD-B-responsible sites might be between -147 and -85 region of the ATF3 promoter. EAFAD-B-induced ATF3 promoter activity was significantly decreased when the CREB site was deleted. However, the deletion of Ftz sites did not affect ATF3 promoter activity by EAFAD-B. We also observed that inhibition of p38MAPK and GSK3β attenuated EAFAD-B-mediated ATF3 promoter activation. Also, EAFAD-B contributes at least in part to increase of ATF3 accumulation.

**Conclusion:**

These findings suggest that the anti-cancer activity of EAFAD-B may be a result of ATF3 promoter activation and subsequent increase of ATF3 expression.

## Background

*Abeliophyllum distichum* Nakai (*A. distichum*) has been known to be a deciduous shrub and native to the south and central areas of Korea [1]. Recently, A. distichum has been reported to exert the inhibitory effect on angiotensin converting enzyme [1]. However, no specific pharmacological effects from *A. distichum* have been described. Therefore, extensive biological screenings of *A. distichum* may be necessary to examine the potential use.

Activating transcription factor 3 (ATF3) as a member of the ATF/CREB family is activated under various physiological and pathological stimuli [2], which has been regarded to exert cell-depending effects including cell cycle arrest and apoptosis [3, 4]. For the incidence and development of cancer, the roles of ATF3 in cancer progression and anti-cancer therapeutics are complex. There are growing evidences to suggest that ATF3 is expressed in cells treated with serum and growth factor and induces DNA synthesis and expression of cyclin D1 in hepatocytes [5, 6]. Furthermore, ATF3 activates the canonical Wnt/β-catenin pathway in human breast cancer cell [7]. However, ATF3 activation promotes a proapoptotic response, as there are evidences to suggest that ATF3 overexpression induced apoptosis in human colorectal cancer cells [8–10]. Therefore, it is suggested that ATF3 activation may be a promising cancer preventive and therapeutic target in human colorectal cancer.

In the course of this investigation, we tested the anti-cancer effect of *A. distichum* in human cancer cell lines and elucidated the potential anti-cancer mechanism of *A. distichum* in human colorectal cancer cells.

## Methods

### Chemicals

Cell culture media, Dulbecco's Modified Eagle medium (DMEM)/F-12 1:1 Modified medium (DMEM/F-12) was purchased from Lonza (Walkersville, MD, USA). 3-(4,5-dimethylthizaol-2-yl)-2,5-diphenyl tetrazolium bromide (MTT) were purchased from Sigma Aldrich (St. Louis, MO, USA). SB203580, PD98059 were purchased from Calbiochem (San Diego, CA, USA). SB216763 were purchased from Sigma Aldrich. ATF3 antibody and ATF3 siRNA were purchased from Santa Cruz Biotechnology, Inc (Santa Cruz, CA, USA). Antibodies against β-actin, Poly (ADP-ribose) polymerase (PARP), p38, p-p38, ERK1/2, p-ERK1/2, p-GKS3β and GKS3β were purchased from Cell Signaling (Bervely, MA, USA). ATF3 promoter constructs (-1420/+34, -718/+34, -514/+34, -318/+34, -147/+34 and -84/+34, pATF3-514 del Ftz and pATF3-514 del CRE) were kindly provided by Dr. S-H Lee (University of Maryland College Park, Maryland, USA). All chemicals were purchased from Fisher Scientific, unless otherwise specified.

### Sample preparation

The plant parts of *A. distichum* (voucher number: Park1001(ANH)) was formally identified by Jae Ho Park as the professor of Jungwon University, Korea. Its flower, leaf and branch were cultivated and collected at Goesan-gun, Chungbuk, Korea. Five hundred grams of fresh parts was extracted with 1,000 ml of 80% methanol with shaking for 24 h. The methanol-soluble fraction was filtered and concentrated to approximately 20 ml volume using a vacuum evaporator and a fraction was placed in a separating funnel. The ethyl acetate fractions from the parts of *A. distichum* was separated from the mixture, evaporated by a vacuum evaporator and prepared aseptically and kept in a refrigerator until use.

### Cell culture and treatment

Human colorectal cancer cell lines (HCT116, SW480 and LoVo cells), human breast cancer cell lines (MCF-7 and MDA-MB-231), hepatocellular carcinoma cells (HepG-2) and colon normal cells (CCD-18co) were purchased Korean Cell Line Bank (Seoul, Korea) and grown in DMEM/F-12 supplemented with 10% fetal bovine serum (FBS), 100 U/ml penicillin and 100 μg/ml streptomycin. The cells were maintained at 37°C under a humidified atmosphere of 5% CO_2_. Ethyl acetate fractions from *A. distichum* were dissolved in dimethyl sulfoxide (DMSO) and treated to cells. DMSO was used as a vehicle and the final DMSO concentration was not exceeded 0.1% (v/v).

### Cell viability

Cell viability was measured using MTT assay system. Briefly, cells were plated onto 96-well plated and grown overnight. The cells were treated with 0, 50, 100 and 200 μg/ml of EAFAD-B for 24 and 48 h. Then, the cells were incubated with 50 μl of MTT solution (1 mg/ml) for the additional 2 h. The resulting crystals were dissolved in DMSO. The formation of formazan was measured by reading absorbance at a wavelength of 570 nm.

### Reverse transcriptase-polymerase chain reaction (RT-PCR)

Total RNA was prepared using a RNeasy Mini Kit (Qiagen, Valencia, CA, USA) and total RNA (1 μg) was reverse-transcribed using a Verso cDNA Kit (Thermo Scientific, Pittsburgh, PA, USA) according to the manufacturer’s protocol for cDNA synthesis. PCR was carried out using PCR Master Mix Kit (Promega, Madison, WI, USA) with primers for human ATF3 and human GAPDH as follows: human ATF3: 5′-gtttgaggattttgctaacctgac-3′, and reverse 5′-agctgcaatcttatttctttctcgt-3′; huaman GAPDH: forward 5’-acccagaagactgtggatgg-3’ and reverse 5’-ttctagacggcaggtcaggt-3’.

### Transient transfections

Transient transfections were performed using the PolyJet DNA transfection reagent (SignaGen Laboratories, Ijamsville, MD, USA) according to the manufacturers’ instruction. HCT116 and SW480 cells were plated in 12-well plates at a concentration of 2 × 10^5^ cells/well. After growth overnight, plasmid mixtures containing 0.5 μg of ATF3 promoter linked to luciferase and 0.05 μg of *pRL-null* vector were transfected for 24 h. The transfected cells were cultured in the absence or presence of the ethyl acetate fractions for the indicated times. The cells were then harvested in 1 × luciferase lysis buffer, and luciferase activity was normalized to the *pRL-null* luciferase activity using a dual-luciferase assay kit (Promega).

### Transfection of small interference RNA (siRNA)

The cells were plated in six-well plates and incubated overnight. HCT116 cells were transfected with control siRNA and ATF3 siRNA for 48 h at a concentration of 100 nM using TransIT-TKO transfection reagent (Mirus, Madison, WI) according to the manufacturer's instruction. Then the cells were treated with EAFAD-B (200 μg/ml) for 24 h.

### SDS-PAGE and Western blot

After EAFAD-B treatment, cells were washed with 1 × phosphate-buffered saline (PBS), and lysed in radioimmunoprecipitation assay (RIPA) buffer (Boston Bio Products, Ashland, MA, USA) supplemented with protease inhibitor cocktail (Sigma Aldrich) and phosphatase inhibitor cocktail (Sigma Aldrich), and centrifuged at 15,000 × g for 10 min at 4°C. Protein concentration was determined by the bicinchoninic acid (BCA) protein assay (Pierce, Rockford, IL, USA). The proteins were separated on SDS-PAGE and transferred to PVDF membrane (Bio-Rad Laboratories, Inc., Hercules, CA, USA). The membranes were blocked for non-specific binding with 5% nonfat dry milk in Tris-buffered saline containing 0.05% Tween 20 (TBS-T) for 1 h at room temperature and then incubated with specific primary antibodies in 5% nonfat dry milk at 4°C overnight. After three washes with TBS-T, the blots were incubated with horse radish peroxidase (HRP)-conjugated immunoglobulin G (IgG) for 1 h at room temperature and chemiluminescence was detected with ECL Western blotting substrate (Amersham Biosciences) and visualized in Polaroid film.

### Statistical analysis

Statistical analysis was performed with the Student's unpaired *t*-test, with statistical significance set at *, P < 0.05.

## Results

### *A. distichum*induces reduction of cell viability against various cancer cells

**Figure 1:**
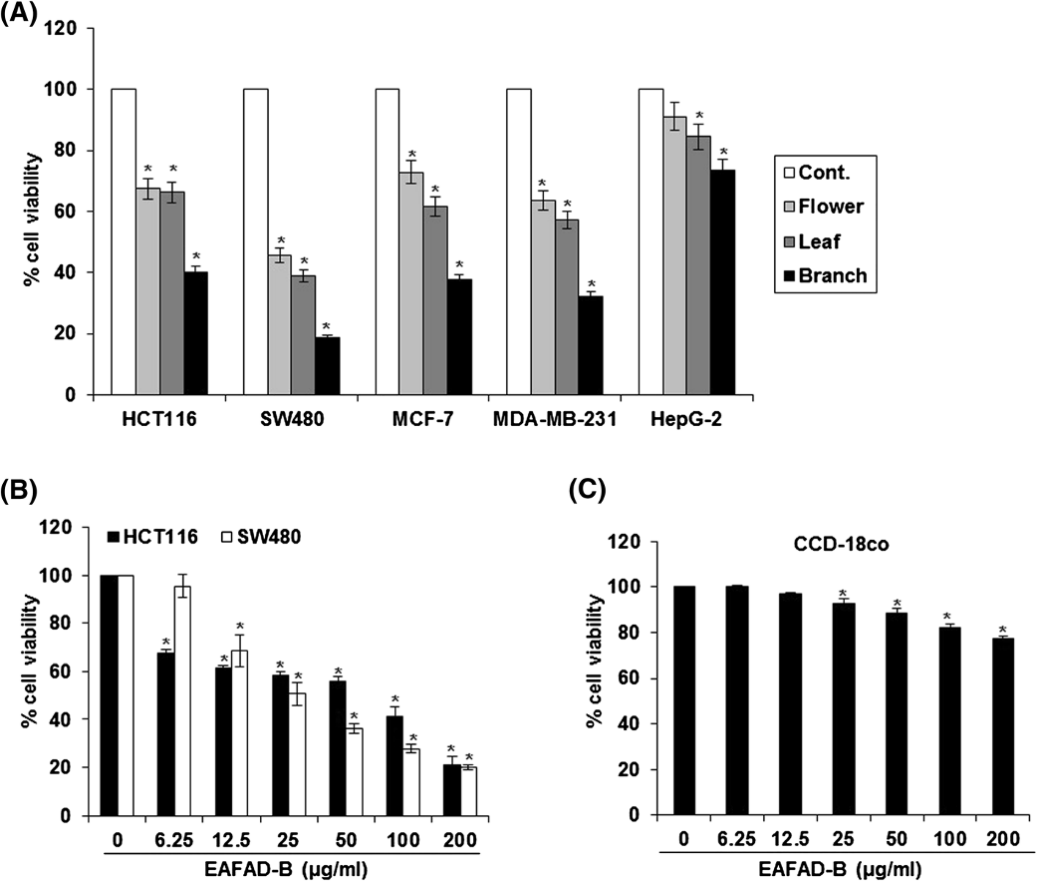
**Effect of EAFAD on cell viability in a variety of cancer cells and normal colon cells. (A,B)** Human colorectal cancer cells (HCT116 and SW480), breast cancer cells (MCF-7 and MDA-MB-231), hepatocellular carcinoma cells (HepG-2) and **(C)** normal colon cells (CCD-18co) were plated overnight and then each fraction was treated for 24 h. Cell viability was measured using MTT assay as described in Materials and methods. *P < 0.05 compared to cells without EAFAD treatment.

The effects of the ethyl acetate fraction from the parts of *A. distichum* on cell viability were evaluated in human breast cancer cells (MCF-7 and MDA-MB-231), colorectal cancer cells (HCT116 and SW480), hepatocellular carcinoma cells (HepG-2) and colon normal cells (CCD-18co) by MTT assay. As shown in Figure 1A, cell viability of all cancer cells was reduced by the treatment of 100 μg of the ethyl acetate fraction from the parts of *A. distichum* for 24 h and this effect was highest in the ethyl acetate fraction from the branch of *A. distichum.* There, we collected the ethyl acetate fraction from the branch of *A. distichum* (EAFAD-B) for the further studies elucidating the potential mechanism by which *A. distichum* exerts anti-cancer activity*.* In a dose-experiment for evaluating the effect of EAFAD-B on cell viability, EAFAD-B dose-dependently reduced the cell viability of human colorectal cancer cells (Figure 1B). However, EAFAD-B induced minimal reduction of cell viability in CCD-18co (colon normal cells) (Figure 1C). These data indicate that EAFAD-B’s anti-cancer activity may be cancer-specific.

### Effect of EAFAD-B on ATF3 expression and ATF3-mediated apoptosis in human colorectal cancer cells

**Figure 2:**
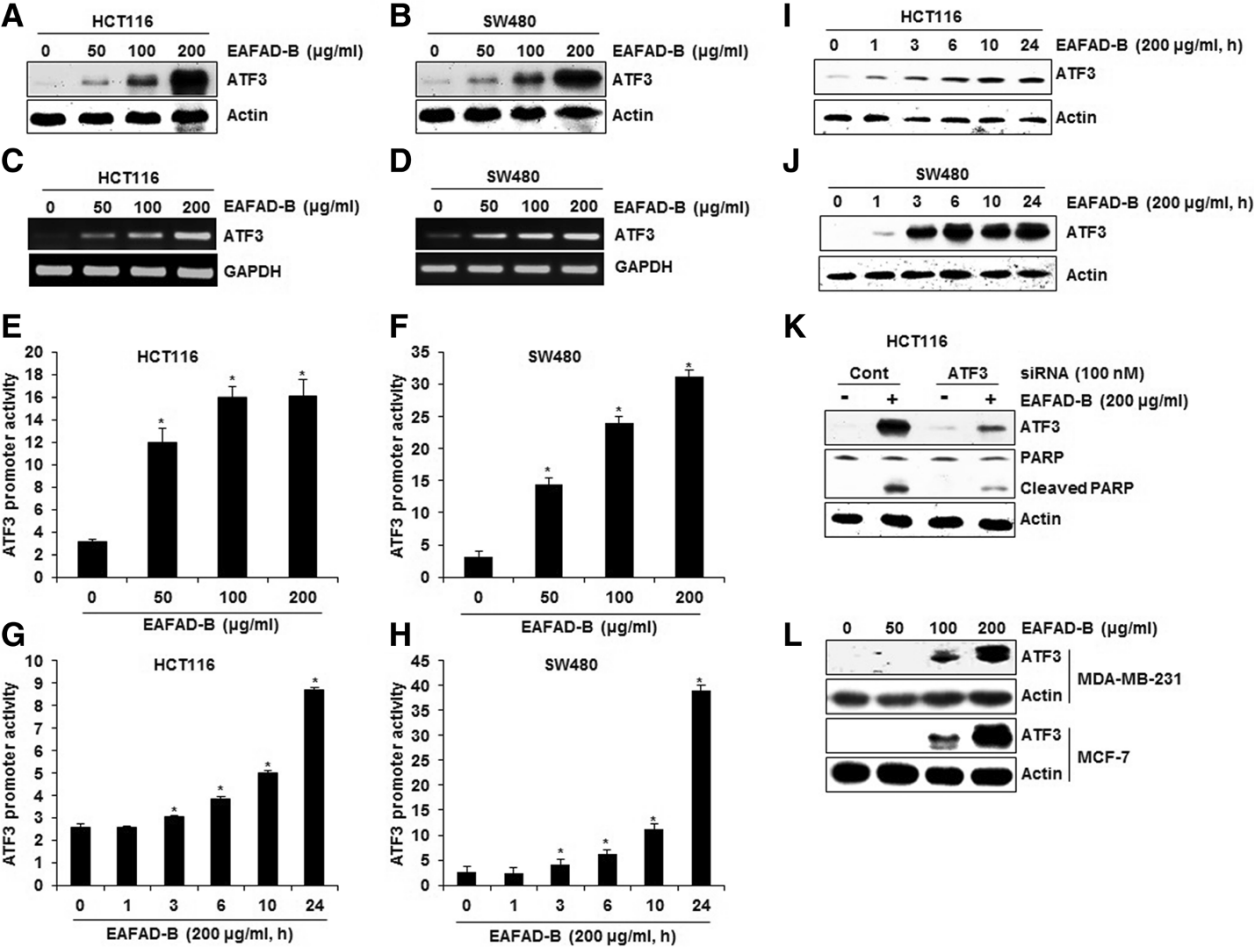
**Effect of EAFAD-B on ATF3 expression and ATF3-mediated apoptosis in human colorectal cancer cells. (A, B, I, J)** HCT116 and SW480 cells were plated and then treated with EAFAD-B. Cell lysates were subjected to SDS-PAGE the Western blot was performed using antibodies against ATF3. Actin was used as internal control. **(C, D)** RT-PCR analysis of ATF3 gene expression, total RNA was prepared after EAFAD-B treatment for 24 h. GAPDH was used as internal control **(E, F, G, H)** For ATF3 promoter activity, luciferase construct containing -1420 to +34 of human ATF3 promoter region was cotransfected with *pRL-null* vector and the cells were treated with EAFAD-B and luciferase activity was measured. *P < 0.05 compared to cells without EAFAD-B treatment. **(K)** ATF3 siRNA was transfected into HCT116 for 48 h and then EAFAD-B was treated for 24 h. Cell lysates were subjected to SDS-PAGE the Western blot was performed using antibodies against PARP. Actin was used as internal control.

There is a growing body of evidence that ATF3 expression could induce apoptosis in human colorectal cancer cells. To test whether EAFAD-B affects ATF3 expression, we measured ATF3 expression in HCT116 and SW480 cells by Western blot and RT-PCR. As shown in Figure 2A and B, ATF3 expression was activated in EAFAD-B-treated HCT116 and SW480 cells. In addition, to elucidate a molecular mechanism by which EAFAD-B increases ATF3 expression in HCT116 and SW480 cells, we first measured the mRNA level of ATF3 to determine whether increase in ATF3 protein expression is involved in transcriptional regulation. ATF3 mRNA was increased in HCT116 and SW480 cells treated with EAFAD-B at the dose-dependent manner (Figure 2C and D), which was similar to protein expression. In addition, we confirmed the transcriptional regulation of ATF3 gene by EAFAD-B using ATF3 promoter luciferase constructs (pATF3-1420/+34). As shown in Figure 2E and F, EAFAD-B dose-dependently increased ATF3 promoter activity in HCT116 and SW480 cells. In time-course experiment, ATF3 promoter activity was increased at 3 h after EAFAD-B treatment in both HCT116 and SW480 cells (Figure 2G and H), while, ATF3 protein expression was slightly induced at 1 h after EAFAD-B treatment (Figure 2I and J).To see whether ATF3 expression is associated with EAFAD-B-mediated apoptosis, cleaved PARP was measured by Western blot in ATF3 knock downed-HCT116 cells. As shown in Figure 2K, knockdown of ATF3 by ATF3 siRNA reduced the cleavage of PARP, indicating that ATF3 may be one of important genes in apoptosis by EAFAD-B.

### Involvement of CREB in EAFAD-B-induced ATF3 activation

**Figure 3:**
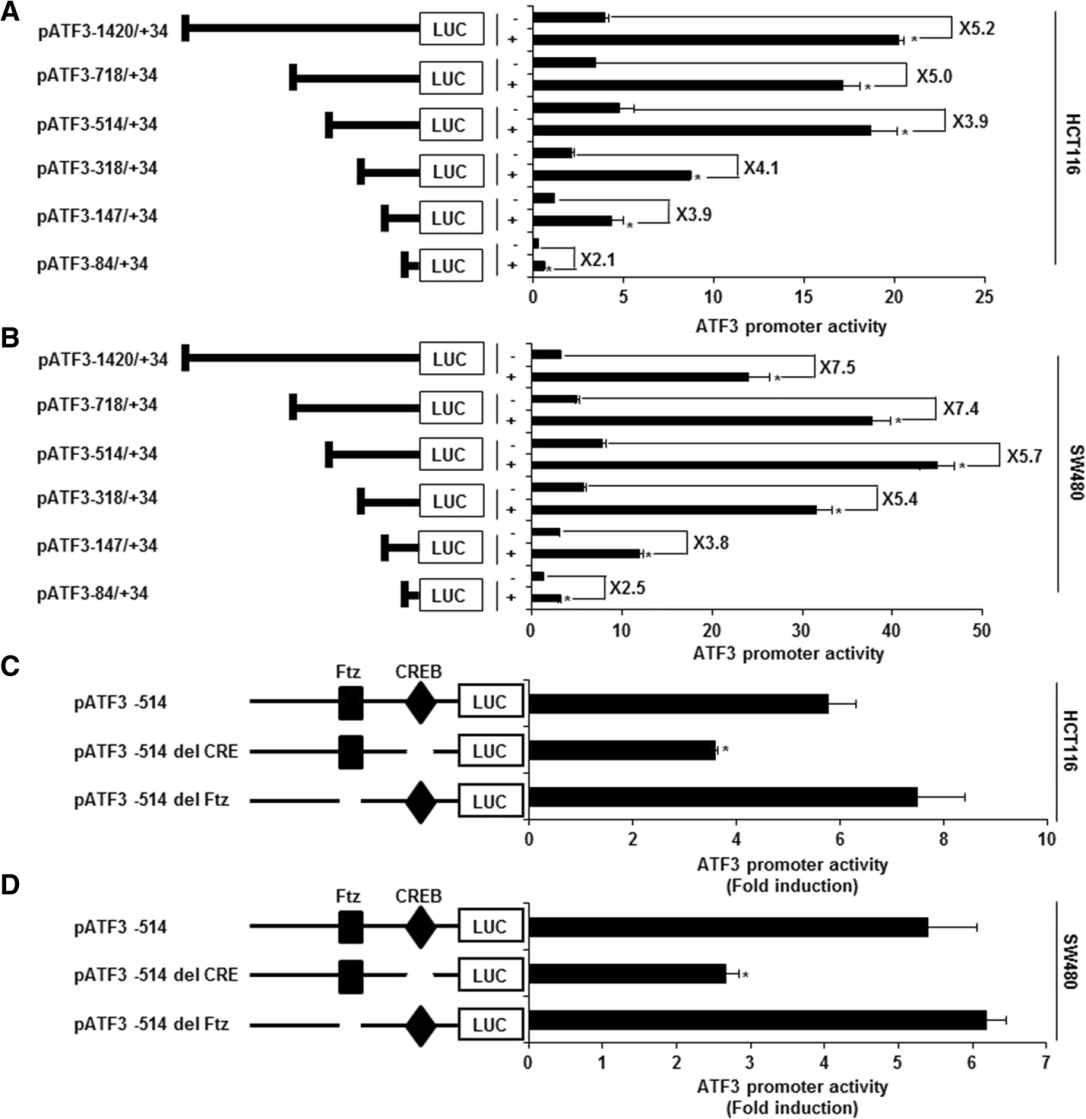
**Identification of ATF3 promoter sites responsible for EAFAD-B-induced ATF3 activation. (A, B)** Each indicated constructs or **(C, D)** each deletion construct (0.5 μg) of the ATF3 promoter (0.5 μg) was co-transfected with 0.05 μg of *pRL-null* vector into HCT116 and SW480 cells, and cells were treated with 200 μg/ml of EAFAD-B. Luciferase activity was measured. *P < 0.05 compared to cells without EAFAD-B treatment.

To elucidate a specific site of ATF3 promoter region associated with promoter activation by EAFAD-B, ATF3 promoter luciferase constructs (pATF3-1420/+34, pATF3-718/+34, pATF3-514/+34, pATF3-318/+34, pATF3-147/+34 and pATF3-84/+34) were transfected into HCT116 and SW480 cells and treated with 200 μg/ml of EAFAD-B for 24 h. As shown in Figure 3A and B, the fold inductions in HCT116 were 5.2, 5.0, 3.9, 4.1, 3.9 and 2.1 in pATF3-1420/+34, pATF3-718/+34, pATF3-514/+34, pATF3-318/+34, pATF3-147/+34 and pATF3-84/+34, respectively. In SW480 cells, EAFAD-B activated ATF3 promoter activity by 7.5, 7.4, 5.7, 5.4, 3.8 and 2.5 in pATF3-1420/+34, pATF3-718/+34, pATF3-514/+34, pATF3-318/+34, pATF3-147/+34 and pATF3-84/+34, respectively. EAFAD-B increased ATF3 promoter activity by 4-fold using ATF3 promoter construct containing regions between -1420 and -147, while EAFAD-B slightly increased ATF3 promoter activity using a construct containing the -84/+34 region, which indicates that ATF3 promoter region (-146/-85) may be important for ATF3 activation by EAFAD-B. The Fushi tarazu (Ftz) and CREB have been reported to be *cis*-acting elements in ATF3 promoter containing -147 and -85 [9]. To identify the role of each *cis*-acting element, each site-deleted ATF3 promoter constructs were transfected into HCT116 and SW480 cells and and treated with 200 μg/ml of EAFAD-B for 24 h. As shown in Figure 3C, EAFAD-B-induced ATF3 promoter activity was significantly decreased when the CREB site was deleted. However, the deletion of Ftz sites did not affect ATF3 promoter activity by EAFAD-B. These data indicated that CREB is an important region in EAFAD-B induced ATF3 expression.

### ATF3 transcriptional activation by EAFAD-B is dependent on p38 MAPK and GSK3β

**Figure 4:**
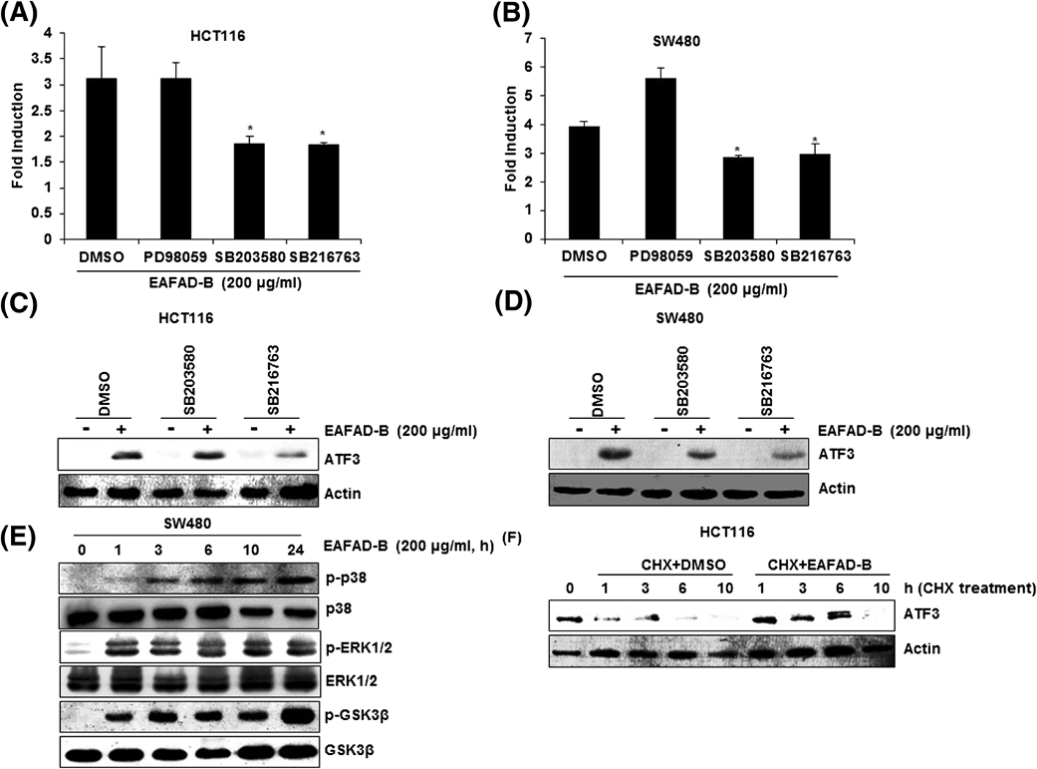
**Up-stream signaling pathways affecting EAFAD-B-mediated ATF3 activation. (A, B)** Luciferase construct containing -1420 to +34 of human ATF3 promoter region was cotransfected with *pRL-null* vector. Then, the cells were pretreated with 20 μM of PD98059 (ERK1/2 inhibitor), SB203580 (p38 inhibitor) or SB216763 (GSK3β inhibitor) and then co-treated with 200 μg/ml of EAFAD-B for 24 h. Luciferase activity was measured. *P < 0.05 compared to cells without EAFAD-B treatment. **(C, D)** HCT116 and SW480 cells were pre-treated with 20 μM of PD98059 (ERK1/2 inhibitor), SB203580 (p38 inhibitor) or SB216763 (GSK3β inhibitor) and then co-treated with 200 μg/ml of EAFAD-B for 24 h. Cell lysates were subjected to SDS-PAGE the Western blot was performed using antibodies against ATF3. Actin was used as internal control. **(E)** SW480 cells were treated with 200 μg/ml of EAFAD-B for indicated times. Cell lysates were subjected to SDS-PAGE and the Western blot was performed using antibodies against p-p38, p38, p-ERK1/2, ERK1/2, p-GSK3β or GSK3β. **(F)** HCT116 cells were pre-treated with 200 μg/ml of EAFAD-B for 12 h and then co-treated with CHX (10 μg/ml) for the indicated times in absence/presence of EFAD-B. Cell lysates were subjected to SDS-PAGE and the Western blot was performed using antibodies against ATF3. Actin was used as internal control.

To determine the upstream kinase affecting EAFAD-B-induced ATF3 transcriptional activation, ATF3 promoter construct (pATF3-1420/+34) was transfected into HCT116 and SW480 cells and then these cells were pretreated with 20 μM of PD98059 (ERK1/2 inhibitor), SB203580 (p38 inhibitor) or SB216763 (GSK3β) for 2 h and subsequently co-treated with 200 μg/ml of EAFAD-B for 24 h. As shown in Figure 4A and B, inhibitions of p38 MAPK by SB203580 and GSK3β by SB216763 attenuated EAFAD-B-induced ATF3 transcriptional activation. However, inhibition of ERK1/2 by PD98059 did not affect ATF3 activation by EAFAD-B. We also tested that the inhibitions of p38MAPK and GSK3β affect ATF3 protein level. In these experiments, the inhibition of GSK3β by SB216763 inhibited the increase of ATF3 protein level by EAFAD-B, while p38 MAPK inhibition did not affect ATF3 protein level in HCT116 cells (Figure 4C). However, the inhibitions of p38MAPK and GSK3β reduced EAFAD-B-induced ATF3 protein expression in SW480 cells (Figure 4D). To see whether EAFAD-B affects the activation of p38, ERK1/2 and GSK3β, we measured the phosphorylation level of these kinases by Western blot and found that EAFAD-B increased the phosphorylation of three kinases from 1 h after the treatment (Figure 4E).Interestingly, our findings indicate that inhibition of p38 MAPK differently affect ATF3 transcriptional activation and increase of ATF3 protein in HCT116 cells. Moreover, the time point to increase ATF3 protein level (1 h after EAFAD-B treatment) is earlier than that to start ATF3 transcriptional activation (3 h after EAFAD-B treatment) (Figure 2G-J). Thus, we hypothesize that EAFAD-B-induced increase of ATF3 protein could be consequence of ATF3 accumulation. To address this question, we investigated the effect of EAFAD-B on ATF3 stability in HCT116 cells. As shown in Figure 4F, the treatment of cycloheximide (CHX) after pre-stimulation of EAFAD-B for 12 h decreased ATF3 protein level. However, co-treatment of EAFAD-B with CHX attenuated CHX-induced decrease of ATF3 protein level. In conclusion, these results demonstrate that EAFAD-B increased the transcription activation and increase of ATF3 protein level through p38 and GSK3β activation and also EAFAD-B contributes at least in part to increase of ATF3 accumulation.

## Discussion

Cancer chemoprevention has received attention as the most effective approach to reduce colorectal cancer related mortality. *Abeliophyllum distichum* Nakai (*A. distichum*) has been known to be a deciduous shrub and native to the south and central areas of Korea [1]. Recently, *A. distichum* has been reported to exert the inhibitory effect on angiotensin converting enzyme [1]. However, no specific pharmacological effects from *A. distichum* have been described. In search for approaches to augment the potential therapeutic efficacy of *A. distichum* for human colorectal cancer, we investigated the anticancer activity of *A. distichum* and the role of ATF3 in apoptosis by *A. distichum* in human colorectal cancer cells. In this study, we for the first time provide evidence that *A. distichum* exerts anti-cancer effect, which especially is associated with ATF3 activation in human colorectal cancer cells.

The activation of activating transcription factor 3 (ATF3) has been known to exert a proapoptotic effect in human colorectal cancer [8–10] and ATF3 is a major target of some anticancer drugs including cisplatin [11] and bortezomib [12]. In addition, there is a growing body of evidences to suggest that antitumorigenic phytochemicals induce ATF3-mediated apoptosis [13–15]. This indicates that ATF3 may be an important molecular target of cancer chemoprevention against human colorectal cancer. Our data show that the ethyl acetate fraction from the branch of *A. distichum* (EAFAD-B) increased ATF3 expression and ATF3 knockdown by siRNA attenuated apoptosis by EAFAD-B, which indicates that ATF3 may be one of the molecular targets for anti-cancer activity of EAFAD-B in human colorectal cancer cells.

ATF3 is both tumor suppressive in early stage tumorigenesis and oncogenic in late stage tumorigenesis in breast cancer cell lines [16]. Although ATF3 has been well known to promote metastasis in the breast cancer [17], ATF3 expression can induce apoptosis in human breast cancer cells (MCF-7 and MDA-MB-231) [18, 19]. In addition, ATF3 has been reported to mediate the suppression of cell viability in human hepatocellular carcinoma cells (HepG-2) [20]. In this study, EAFAD-B reduced the cell viability in MCF-7, MDA-MB-231 and HepG-2 cells. Thus, the further elucidation of mechanisms for ATF3 role involved in EAFAD-B-induced apoptosis may be needed in human breast cancer and hepatocellular carcinoma cells.

The level of ATF3 can be regulated by multiple mechanisms. One is through transcriptional regulation. In this study, our data shows that EAFAD-B activated the expression of ATF3 mRNA through increasing promoter activity, which indicates upregulation of ATF3 by EAFAD-B may be dependent on transcription. Another mechanism to regulating ATF3 level is through suppression of proteasomal degradation although it is generally believed that ATF3 induction is activated mainly through transcriptional regulation [21]. There is growing evidence that MDM2 induces ubiquitination and subsequent proteasomal degradation of ATF3 [22]. In this study, we found two evidences that EAFAD-B may affect ATF3 stability. One is that EAFAD-B increased the protein level of ATF3 at earlier time than mRNA level. Another evidence is that p38 inhibition attenuated ATF3 promoter activation by EAFAD-B, while did not affect ATF3 protein level. Furthermore, we found that CHX treatment time-dependently decreased ATF3 protein level upregulated by the pre-treatment of EAFAD-B, while co-treatment of CHX and EAFAD-B attenuated the reduction of ATF3 protein. These findings indicate that EAFAD-B may inhibit ATF3 proteasomal degradation.

Many transcription factors such as NF-κB, EGR-1, E2F, AP-1, CREB and Ftz have been identified to regulate ATF3 transcriptional activity [23]. We determined that EAFAD-B-responsible sites for ATF3 transcriptional activity might be between the -147 and -85 region in ATF3 promoter because ATF3 promoter activity by EAFAD-B was more decreased in transfection of ATF3 promoter (-85/+34) than other transfection such as pATF3-1420/+34, pATF3-718/+34, pATF3-514/+34, pATF3-318/+34, pATF3-147/+34. Especially, CREB and Ftz are cis-acting elements in ATF3 promoter (-147/-85) [9]. To identify the role of each *cis*-acting element affecting EAFAD-B-mediated ATF3 transcriptional activation, each site-deleted ATF3 promoter constructs were transfected into HCT116 and SW480 cells and we found that EAFAD-B-induced ATF3 promoter activity was significantly decreased when the CREB site was deleted. These data indicated that CREB is an important region in EAFAD-B-induced ATF3 expression. However, we do not exclude the effects of other transcription factors because EAFAD-B-mediated ATF3 promoter activity was gradually decreased in pATF3-1420/+34, pATF3-718/+34, pATF3-514/+34, pATF3-318/+34, pATF3-147/+34 and pATF3-84/+34. Thus, the further experiments for more investigation of the transcription factors associated with EAFAD-B-induced ATF3 activation may be needed. We also observed the basal activity of ATF3 promoter was different in ATF3 promoter constructs. We think that ATF3 promoter activity can be affected by other transcriptional activity such as Sp3, NF-kB and c-Jun. Indeed, Sp3 and c-Jun have been reported to increase basal ATF3 promoter activity [9, 24].

It has been reported that ATF3 expression is regulated by MAPK signaling [15, 25] and GSK3β [14]. So, we examined whether EAFAD-B-mediated ATF3 activation is associated with the activation of ERK1/2, p38 or GSK3β and found that EAFAD-B induced the phosphorylation of ERK1/2, p38 or GSK3β and inhibition of p38 or GSK3β attenuated ATF3 promoter activity by EAFAD-B in HCT116 and SW480 cells. However, ERK1/2 inhibition did not affect ATF3 promoter activity. These findings indicate that ATF3 activation by EAFAD-B may be dependent on p38 or GSK3β but not ERK1/2. However, we do not excluded that the EAFAD-B-induced ATF3 activation may be overcome by ERK1/2 inhibitor.

## Conclusions

These findings highlight that EAFAD-B increased the transcription activation and increase of ATF3 protein level through p38 and GSK3β activation and also EAFAD-B contributes at least in part to increase of ATF3 accumulation. In conclusion, the current study provides information on the anti-cancer activity and the potential molecular mechanism of *A. distichum*.

## Abbreviations

ATF3: Activating transcription factor 3
EAFAD-B: Ethyl acetate fraction from the branch of *A. distichum*.

## References

[1] Oh H, Kang DG, Kwon TO, Jang KK, Chai KY, Yun YG, Chung HT, Lee HS (2003). Four glycosides from the leaves of Abeliophyllum distichum with inhibitory effects on angiotensin converting enzyme. Phytother Res.

[2] Hai T, Hartman MG (2001). The molecular biology and nomenclature of the activating transcription factor/cAMP responsive element binding family of transcription factors: activating transcription factor proteins and homeostasis. Gene.

[3] Cai Y, Zhang C, Nawa T, Aso T, Tanaka M, Oshiro S, Ichijo H, Kitajima S (2000). Homocysteine-responsive ATF3 gene expression in human vascular endothelial cells: activation of c-Jun NH(2)-terminal kinase and promoter response element. Blood.

[4] Yin T, Sandhu G, Wolfgang CD, Burrier A, Webb RL, Rigel DF, Hai T, Whelan J (1997). Tissue-specific pattern of stress kinase activation in ischemic/reperfused heart and kidney. J Biol Chem.

[5] Iyer VR, Eisen MB, Ross DT, Schuler G, Moore T, Lee JC, Trent JM, Staudt LM, Hudson J, Boguski MS, Lashkari D, Shalon D, Botstein D, Brown PO (1999). The transcriptional program in the response of human fibroblasts to serum. Science.

[6] Allan AL, Albanese C, Pestell RG, LaMarre J (2001). Activating transcription factor 3 induces DNA synthesis and expression of cyclin D1 in hepatocytes. J Biol Chem.

[7] Yan L, Della Coletta L, Powell KL, Shen J, Thames H, Aldaz CM, MacLeod MC (2011). Activation of the canonical Wnt/beta-catenin pathway in ATF3-induced mammary tumors. PLoS One.

[8] Yamaguchi K, Lee SH, Kim JS, Wimalasena J, Kitajima S, Baek SJ (2006). Activating transcription factor 3 and early growth response 1 are the novel targets of LY294002 in a phosphatidylinositol 3-kinase-independent pathway. Cancer Res.

[9] Cho KN, Sukhthankar M, Lee SH, Yoon JH, Baek SJ (2007). Green tea catechin (-)-epicatechin gallate induces tumour suppressor protein ATF3 via EGR-1 activation. Eur J Cancer.

[10] Lee SH, Min KW, Zhang X, Baek SJ (2013). 3,3'-diindolylmethane induces activating transcription factor 3 (ATF3) via ATF4 in human colorectal cancer cells. J Nutr Biochem.

[11] St Germain C, Niknejad N, Ma L, Garbuio K, Hai T, Dimitroulakos J (2010). Cisplatin induces cytotoxicity through the mitogen-activated protein kinase pathways and activating transcription factor 3. Neoplasia.

[12] Bruning A, Burger P, Vogel M, Rahmeh M, Friese K, Lenhard M, Burges A (2009). Bortezomib treatment of ovarian cancer cells mediates endoplasmic reticulum stress, cell cycle arrest, and apoptosis. Investig New Drugs.

[13] Lee SH, Kim JS, Yamaguchi K, Eling TE, Baek SJ (2005). Indole-3-carbinol and 3,3'-diindolylmethane induce expression of NAG-1 in a p53-independent manner. Biochem Biophys Res Commun.

[14] Lee SH, Yamaguchi K, Kim JS, Eling TE, Safe S, Park Y, Baek SJ (2006). Conjugated linoleic acid stimulates an anti-tumorigenic protein NAG-1 in an isomer specific manner. Carcinogenesis.

[15] Baek SJ, Kim JS, Jackson FR, Eling TE, McEntee MF, Lee SH (2004). Epicatechin gallate-induced expression of NAG-1 is associated with growth inhibition and apoptosis in colon cancer cells. Carcinogenesis.

[16] Yin X, Dewille JW, Hai T (2008). A potential dichotomous role of ATF3, an adaptive-response gene, in cancer development. Oncogene.

[17] Dong Y, Asch HL, Ying A, Asch BB (2002). Molecular mechanism of transcriptional repression of gelsolin in human breast cancer cells. Exp Cell Res.

[18] Niknejad N, Gorn-Hondermann I, Ma L, Zahr S, Johnson-Obeseki S, Corsten M, Dimitroulakos J (2014). Lovastatin-induced apoptosis is mediated by activating transcription factor 3 and enhanced in combination with salubrinal. Int J Cancer.

[19] Colin-Cassin C, Yao X, Cerella C, Chbicheb S, Kuntz S, Mazerbourg S, Boisbrun M, Chapleur Y, Diederich M, Flament S, Grillier-Vuissoz I (2013). PPARgamma-inactive Delta2-troglitazone independently triggers ER stress and apoptosis in breast cancer cells. Mol Carcinog.

[20] Lee YM, Uhm KO, Lee ES, Kwon J, Park SH, Kim HS (2008). AM251 suppresses the viability of HepG2 cells through the AMPK (AMP-activated protein kinase)-JNK (c-Jun N-terminal kinase)-ATF3 (activating transcription factor 3) pathway. Biochem Biophys Res Commun.

[21] Hai T, Wolfgang CD, Marsee DK, Allen AE, Sivaprasad U (1999). ATF3 and stress responses. Gene Expr.

[22] Mo P, Wang H, Lu H, Boyd DD, Yan C (2010). MDM2 mediates ubiquitination and degradation of activating transcription factor 3. J Biol Chem.

[23] Liang G, Wolfgang CD, Chen BP, Chen TH, Hai T (1996). ATF3 gene. Genomic organization, promoter, and regulation. J Biol Chem.

[24] Fu L, Kilberg MS (2013). Elevated cJUN expression and an ATF/CRE site within the ATF3 promoter contribute to activation of ATF3 transcription by the amino acid response. Physiol Genomics.

[25] Lee SH, Bahn JH, Whitlock NC, Baek SJ (2010). Activating transcription factor 2 (ATF2) controls tolfenamic acid-induced ATF3 expression via MAP kinase pathways. Oncogene.

[CR26] The pre-publication history for this paper can be accessed here:http://www.biomedcentral.com/1472-6882/14/487/prepub

